# Tanshinone IIA ameliorates myocardial ischemia/reperfusion injury in rats by regulation of NLRP3 inflammasome activation and Th17 cells differentiation

**DOI:** 10.1590/acb370701

**Published:** 2022-10-28

**Authors:** Dan Li, Zhihui Yang, Shan Gao, Hao Zhang, Guanwei Fan

**Affiliations:** 1 Master. First Teaching Hospital of Tianjin University of Traditional Chinese Medicine – National Clinical Research Center for Chinese Medicine Acupuncture and Moxibustion – Tianjin, China.; 2 Bachelor. First Teaching Hospital of Tianjin University of Traditional Chinese Medicine – National Clinical Research Center for Chinese Medicine Acupuncture and Moxibustion – Tianjin, China.; 3 PhD. First Teaching Hospital of Tianjin University of Traditional Chinese Medicine – National Clinical Research Center for Chinese Medicine Acupuncture and Moxibustion – Tianjin, China.

**Keywords:** Inflammation, Myocardial Ischemia, Reperfusion Injury, NLR Family, Pyrin Domain-Containing 3 Protein

## Abstract

**Purpose:**

Tanshinone IIA is a well-known lipophilic active constituent refined from traditional Chinese medicines, danshen. It has been previously demonstrated to possess various biological properties, including anti-inflammatory, antioxidant, promoting angiogenesis effect and so on. However, the mechanism of tanshinone IIA on myocardial ischemia-reperfusion injury (MI/RI) remains unclear. In this study, we investigated the effect of tanshinone IIA on MI/RI.

**Methods:**

MI/RI rat models were set up. Echocardiographic evaluation and hematoxylin and eosin staining were performed to analyze the cardiac function and morphology of MI/RI. Western blot was conducted for the detection of protein expression of pyrin domain containing 3 (NLRP3) and caspase-1 in heart tissues. Flow cytometry and real-time polymerase chain reaction were used for the detection of proinflammatory cytokines and Th17 cells differentiation.

**Results:**

We found that tanshinone IIA alleviated the myocardial damage of MI/RI, ameliorated the overall and local inflammatory reaction, and produced a cardioprotective effect by inhibiting of NLRP3 inflammasome activation and Th17/Treg cells differentiation.

**Conclusions:**

Our results highlighted the cardio-protective effect of tanshinone IIA on MI/RI and uncovered its underlying mechanism related to the NLRP3 inflammasome inhibition and the modulation of Th17/Treg cells differentiation.

## Introduction

Acute myocardial infarction (AMI) is one of the most important causes of death in the world, leading to a huge health and economic burden to the world^1^. Rapid recovery of the coronary flow is the most effective strategy to solve the problem of coronary blockage and improve cardiac function. However, rapid reperfusion may aggravate myocardial injury and cause myocardial ischemia/reperfusion injury (MI/RI). The mechanism of MI/RI is complicated. Oxidative stress, calcium overload, neutrophil infiltration and cell apoptosis are all involved in the process of MI/RI.It is still a ticklish problem how to effectively prevent reperfusion injury while recovering the blood flow of ischemic myocardium.

Inflammatory response is involved in the pathophysiological process of MI/RI. The pyrin domain containing 3 (NLRP3) inflammasome consists of NOD-, LRR- and NLRP3, apoptosis-associated speck-like protein containing a caspase recruitment domain (ASC), and pro-caspase-1. When stimulated by pathogens or danger signals, NLRP3 was activated and recruited pro-caspase-1 through ASC, made NLRP3 inflammasome assembly. Then, pro-caspase-1 was activated to be caspase-1. Caspase-1 is a converting enzyme, and it can cleave pro-interleukin-1β (IL-1β) and pro-interleukin-18 (IL-18) into their active forms, which result in the releasing of IL-1β and IL-18, eventually triggering a strong inflammatory response^2^. The mechanisms of NLRP3 inflammasome activation include the generation of reactive oxygen species (ROS), potassium efflux and the release of cathepsins into the cytosol after lysosomal destabilization^2^. During the process of MI/RI, NLRP3-mediated proinflammatory mediators release aggravated the myocardial tissue damage, while NLRP3 deficiency or inhibition improved cardiac function^3^.

Tanshinone IIA is a lipophilic active constituent of the Chinese medicinal herb *Salvia miltiorrhiza* Bge. (danshen). Tanshinone IIA possesses various biological properties, which include anti-inflammatory, antioxidant, promoting angiogenesis effect and so on, and it is mainly used for the treatment of cardio-cerebrovascular system diseases, such as atherosclerosis, myocardial ischemia and arrhythmia^4^^,^ 5. Emerging evidence showed that tanshinone IIA might have the potential activities of anticancer^6^. Studies demonstrated that tanshinone IIA alleviated myocardiocyte apoptosis^7^, cardiac fibroblasts^8^, atherosclerosis^9^, and neuroinflammation^10^.

Our previous studies have shown that tanshinone IIA could inhibit inflammation response and promoted macrophages polarization from pro-inflammatory M1 to anti-inflammatory M2 state^11^, and regulated NLRP3 inflammasome activation in a ROS-NF-κB/P38-NLRP3 pathway in LPS stimulated Raw264.7 cells^12^. However, the molecular mechanism of tanshinone IIA involving MI/RI was still uncertain.

In this study, left anterior descending coronary artery ligation and release were adopted to establish rat MI/RI model. We measured the indexes of the cardiac function, inflammatory response, blood flow dynamics and T cells differentiation, so as to explore the possible role of NLRP3 inflammasome in the process of MI/RI. This study aimed to investigate the characteristics and possible mechanisms of tanshinone IIA on NLRP3 inflammasome activation and T cells immunomodulation, providing potential strategies for the treatment of MI/RI and new experimental evidence for the application of tanshinone IIA.

## Methods

### Myocardial ischemia-reperfusion injury model preparation and drug administration

The study protocol was approved by Tianjin University of Traditional Chinese Medicine Laboratory Animal Ethical Committee. All rats were randomly divided into three groups:

- Sham (n = 10);
- MI/RI model (n = 10);
- MI/RI + tanshinone IIA (n = 10).

After anesthetization, the rats were performed thoracotomy. Then, the left anterior descending coronary artery was ligated using a 6-0 silk suture. After 30 min of ischemia, the ligation was opened and allowed for reperfusion for 2 hours. Rats in sham group underwent the same procedure, but without ligation. For the MI/RI + tanshinone IIA group, tanshinone IIA was administered via gavage with 15 mg/kg for seven consecutive days before the thoracotomies. For the sham and MI/RI model groups, equal amounts of 0.5% carboxymethyl cellulose sodium were administered.

### Echocardiographic assessment of left ventricular function

The left ventricular function was assessed by a Vevo 2100 (VisualSonic, Canada) ultra-high-resolution animal ultrasound imaging system. Ejection fraction (EF%), fractional shortening (FS%) and aortic valve peak velocity were evaluated as the indicators of cardiac function.

### Biochemical analyses of myocardial enzymology

Blood samples were obtained from abdominal aorta 2 hours after surgery. Serum was separated by centrifugation and repackaged in 1.5 mL EP tube and stored in -80 °C for use. The serum level of lactic dehydrogenase (LDH), creatine kinase (CK), and creatine kinase isoenzyme (CK-MB) were measured by an automatic biochemical analyzer.

### ELISA analysis

The content of ROS, IL-18 and IL-1β in serum was determined by enzyme-linked immunoassay (ELISA) kit (YJ028851, ml002816, ml003057) of Shanghai enzyme linked Biotechnology Co.

### 2,3,5-Triphenyltetrazolium chloride staining analysis

The rats were sacrificed 2 hours after reperfusion. The heart samples were collected and divided into six consecutive slices, and then stained with 1% 2,3,5-Triphenyltetrazolium chloride (TTC) for 30 min at 37 °C. Then, the sections were washed with phosphate buffered saline (PBS) and fixed in 4% paraformaldehyde (PFA). After photographed, ImageJ (MD, United States of America) was used to analyze the area of myocardial infarction as previously reported^13^.

### Hematoxylin and eosin staining

The paraffin sections were dewaxed and hydrated, then stained with hematoxylin for 10 min and then rinsed. After differentiated with 0.5% hydrochloric alcohol for 10 s, the sections were incubated with eosin for 5 min. The sections were observed using an optical microscope (Olympus, Japan) for microscopic examination of morphological changes.

### Flow cytometry

Spleens and blood were harvested from the rats, and single-cell suspensions were prepared. Spleens were homogenized using the plunger of a syringe. The single-cell suspensions were collected with a 300-μm cell filter. After centrifugation at 300 × g for 10 min at room temperature, the cell suspensions were resuspended in 3 mL of 1 × PBS. Red blood cell lysis buffer was used to lyse erythrocytes. After washed with 1 × PBS, the cell suspensions were resuspended in 1 × PBS. The final concentration was adjusted to approximately 1 × 10^6^ cells/mL. For the analysis of Th17 cells, the obtained cells were stimulated with PMA/Ionomycin (Multiscience, Hangzhou, China) and Monensin/BFA (Multiscience, Hangzhou, China) for 5 hours. Then, cells were stained with PE-conjugated anti-CD4 antibodies (eBioscience, San Diego, United States of America), permeabilized with FIX&PERM Reagent (Multiscience, Hangzhou, China), and then stained with APC-conjugated anti-IL-17A antibodies (eBioscience, San Diego, United States of America). For measurement of Treg cells, the cells were stained with PerCP-eFluor 710-conjugated anti-CD25 antibodies (eBioscience, San Diego, United States of America) and PE-conjugated anti-CD4 antibodies. A Foxp3 / transcription factor staining buffer set (eBioscience, San Diego, United States of America) was used for subsequent fixation and permeabilization before incubation with eFluor 660-conjugated anti-Foxp3 antibodies (eBioscience, San Diego, United States of America). Flow cytometry was performed with FACS Calibur (BD Biosciences, United States of America). CD4+IL-17A+ cells represented T17 cells, while CD4+CD25+Foxp3+ cells represented Treg cells. Data analysis was performed with the FlowJo software.

### Western blotting

Myocardium tissues were obtained and lysed. Protein concentration was measured using a BCA protein assay kit. Then, protein samples were denatured by heating at 100 °C for 10 min. The protein samples were then separated by SDS-PAGE and transferred onto PVDF membranes. Thereafter, the membranes were incubated with corresponding primary antibodies overnight at 4 °C. The primary antibodies used in this experiment were anti-NLRP3 antibody (Cat. No.15101S, Cell Signaling Technology, 1:1,000), anti-caspase-1 antibody (Cat. No.22915-1-AP, Proteintech, 1:1,000), Caspase-1 Rabbit pAb (Cat. No. A0964, 1:1,000), HRP-conjugated β-actin rabbit mAb (Cat. No. AC028, 1:5,000) and anti-GADPH antibody (Cat. No.10494-1-AP, Proteintech,1:3,000). Then, the PVDF membranes were incubated with corresponding secondary antibody (Cat. No. SAA544Rb19, Cloud-Clone,1:5,000) for 2 hours at room temperature. The protein bands were visualized using ECL Western blotting substrate and photographed using C-DiGit 3600 (Li-Cor, United States of America).

### Real-time quantitative polymerase chain reaction analysis

Total RNA was extracted from tissue or cells using a commercial RNAprep pure kit (#CW0581, CWbio, China) according to manufacturer’s instructions. The isolated RNA was reverse transcribed into cDNA at 42 °C for 50 min and 85 °C for 5 min using a HiFiScript cDNA Synthesis Kit (#CW2569M, CWbio, China). Quantitative polymerase chain reactions (PCR) were performed with the UltraSYBR Mixture (Low ROX) (#CW2601M, CWbio, China) in a Q-PCR instrument (Lightcycler 96, Roche, Switzerland). The PCR protocol was 95 °C for 10 min, followed by 40 cycles of 95 °C for 10 s, 56 for 30 s, and 72 for 32 s. The primers used were shown in Table 1. Fold-change of the target gene mRNA expression was calculated using the 2^−ΔΔCT^ method.

### Isolation of CD4+ T cells

Spleens were harvested from rats of different groups. Single cell preparation was performed from the harvested tissues. Spleens were separately collected in PBS and each one processed by crushing through a 100-μm cell strainer. Crushed spleens were washed with PBS, centrifuged 500 × g for 5 min before red blood cells were lysed in red blood cell lysis buffer (Solarbio, China) for 10 min at room temperature. Lysed cells were washed with PBS, centrifuged, and resuspended in RPMI-1640 (Gibco, United States of America). CD4+T cells were sorted by immunomagnetic selection with MACS CD4 microbeads (Cat. No. 130-090-319, Miltenyi Biotec, Germany) according to the manufacturer’s instructions, and evaluated by flow cytometry on a FACSCalibur (BD biosciences, United States of America).

### siRNA transfection

NLRP3 siRNA or their controls were designed and synthesized by GENEWIZ. CD4+ T cells were transfected with corresponding siRNA, using a Lipofectamine^®^ 2000 (Invitrogen; ThermoFisher Scientific, Inc., United States of America) transfection method according to the manufacturer’s instructions. The sequences of the siRNAs were as follows: si-NLRP3 forward, 5’- GGAACCAGAAGAUCCUAUUTT -3’ and reverse, 5’- AAUAGGAUCUUCUGGUUCCAA -3’. A total of CD4+ T cells (1 × 10^6^) were seeded into 6-well plates. Negative control siRNA (20 μM) and siNLRP3 (20 μM) were transfected into cells. After transfection for 24 h at 37 °C, the cells were harvested.

### Statistical analysis

Data were expressed as the mean ± standard deviation (SD). All the statistical analysis was conducted using GraphPad Software. Statistical significances among groups were assessed by one-way analysis of variance (ANOVA). P < 0.05 was considered statistically significant.

## Results

### Effects of tanshinone IIA on cardiac function

**Figure 1:**
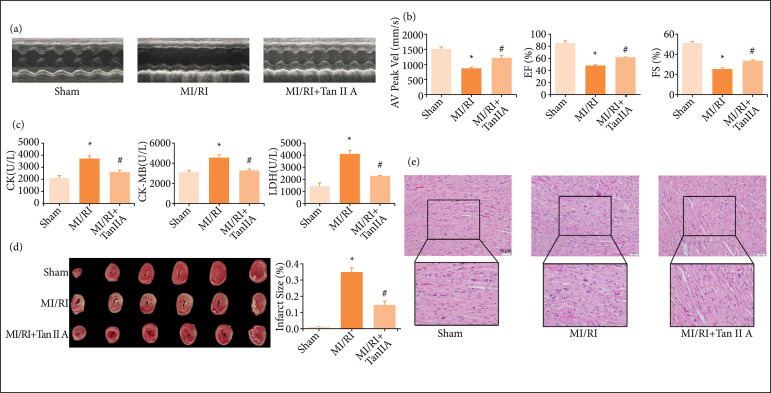
Tanshinone IIA improved cardiac functions against MI/RI. **(a)** Representative echocardiograms for each group. **(b)** Quantification of AV Peak Vel, EF% and FS% (n ≥ 3 per group). **(c)** Levels of CK, CK-MB and LDH in rat serum (n ≥ 3 per group). **(d)** Representative TTC-stained heart images and quantification of infarct sizes. Percentage infarct size =(Infarct size / viable size) × 100% (n ≥ 3 per group). **(e)** Representative HE staining of heart transverse sections (scale bar = 50 μm).

A Vevo 2100 was used to evaluate the effects of tanshinone IIA on cardiac functions. As shown by the echocardiography (Fig. 1a), in the MI/RI model group, the amplitude of left ventricular contraction was decreased compared with the sham group, while the left ventricular contraction movement was enhanced after treatment of tanshinone IIA compared with the MI/RI model group. AV Peak Vel, EF% and FS% were enhanced in the tanshinone IIA group compared with the MI/RI model group (Fig. 1b, p < 0.05), indicating that tanshinone IIA could improve cardiac function after MI/RI. As shown in Fig. 1c, the content of serum CK, CK-MB, and LDH was higher in the MI/RI group compared with the sham group (p < 0.05). However, it decreased significantly after tanshinone IIA treatment compared with model group (Fig. 1c, p < 0.05). These results demonstrated that tanshinone IIA protected cardiac functions against MIRI.

We adopted TTC staining to assess the myocardial infarct size. As shown in Fig. 1d, rats in the model group showed severe myocardial infarction (p < 0.05). In the group of tanshinone IIA treatment, the myocardium infarct volume was decreased significantly (p < 0.05), compared to that in model group. Hematoxylin and eosin (HE) staining was used to evaluate cardiac morphological changes. In sham group, myocardial tissues had normal morphology and orderly arrangement of myocardial fibers. In model group, myocardial tissues exhibited disturbed morphology, disorderly arrangement, and infiltration of inflammatory cells. In tanshinone IIA treatment group, the myocardial injury and inflammatory infiltration were improved compared with those in model group (Fig. 1e).

MI/RI: myocardial ischemia-reperfusion injury; *p < 0.05 compared with the sham group; #p < 0.05 compared with the MI/RI group; TCC: 2,3,5-Triphenyltetrazolium chloride; HE: hematoxylin and eosin.

### Tanshinone IIA attenuated inflammatory response and NLRP3 inflammasome activation

**Figure 2:**
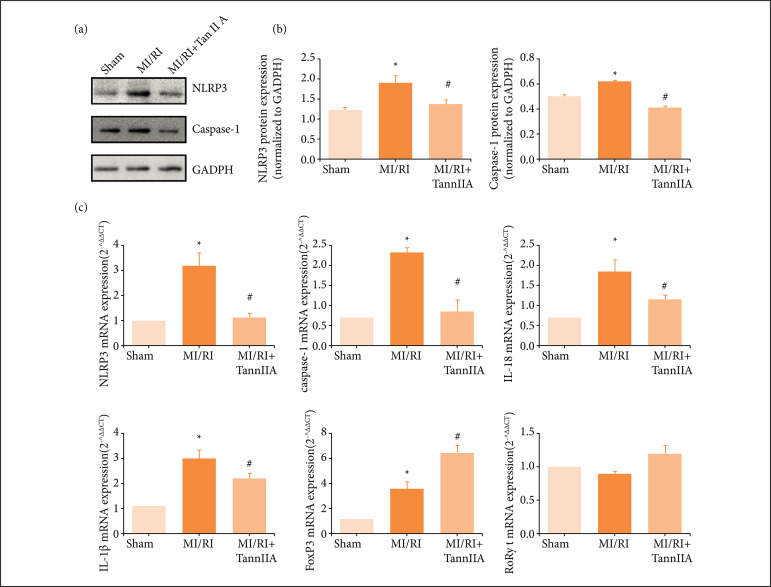
Tanshinone IIA exerted anti-inflammatory activity in rats. **(a)** Representative Western immunoblots for NLRP3 and caspase-1. **(b)** Corresponding quantitative data for Western blotting (n ≥ 3). **(c)** The mRNA expression of NLRP3, caspase-1, IL-18, IL-1β, FoxP3 and RORγt in each group (n ≥ 3).

In the process of MI/RI, NLRP3 inflammasome was regulated by ROS. Then pro-caspase-1 was activated to be caspase-1, which cleaved pro-IL-1β and pro-IL-18 into their active forms, resulting the releasing of IL-1β and IL-18. Suppl. Fig. 1 showed that the content of serum ROS in the model group was increased compared with the sham group. After tanshinone IIA treatment, the content of serum ROS decreased significantly. The data showed that NLRP3 and caspase-1 protein (pro-caspase-1 and cleaved-caspase-1) expression was increased in the model group compared with the sham group, and they were significantly reduced in the treatment groups compared with the model group (Figs. 2a and 2b, and Suppl. Figs. 2a-2c, p < 0.05).

NLRP3: pyrin domain containing 3; MI/RI: myocardial ischemia-reperfusion injury; *P < 0.05 vs. Sham; #P < 0.05 vs. MI/RI; IL: interleukin.

Besides, compared with sham group, the mRNA expression of NLRP3, caspase-1, IL-1β and IL-18 was markedly increased in MI/RI group. After drug treatment, the mRNA expression of NLRP3, caspase-1, IL-1β and IL-18 was decreased markedly (Fig. 2c, p < 0.05). In addition, we also detected the content of IL-18 and IL-1β in serum. Supplementary Fig. 2d showed that the content of IL-18 in serum had been consistent with the mRNA expression result. However, the content of IL-1β was lower than the minimum detection limit of ELISA kit, indicating that its content in serum was very low.

Forkhead box P3 (FoxP3) is a FOXP3 encoding transcription factor, and it programs the function and development of regulatory T cells (Tregs)^14^. The mRNA expression of FoxP3 was markedly increased in MI/RI group, and it increased further in tanshinone IIA treatment group (Fig. 2c, p < 0.05). RORγt has been shown to play a central role in Th17 differentiation^15^^,^ 16. There was no significant difference observed of the mRNA expression of RORγt (Fig. 2c).

### Tanshinone IIA regulated th17/Treg cell balance in MI/RI rats

**Figure 3:**
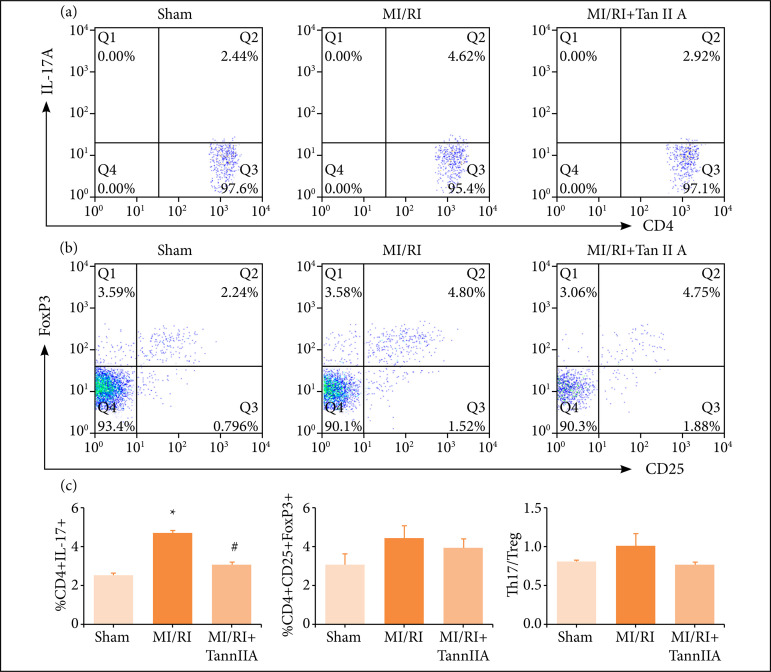
Effect of tanshinone IIA on T cells differentiation in peripheral blood. Representative flow samples of **(a)** CD4+IL17A+ and **(b)** CD4+CD25+FoxP3+ were shown from each sample. **(c)** Quantification of cell frequency of Th17(CD4+IL17A+), Treg (CD4+CD25+FoxP3+) and Th17/Treg (n ≥ 3).

Treg cells are characterized by the co-expression of CD4, CD25 and Foxp3 in the flow cytometry analysis. Th17 cells are characterized by the expression of CD4 and IL-17A. As shown in Figs. 3a-3c, the percentage of Th17 cells were significantly increased in the blood of MI/RI group rats than those in the sham group (Fig. 3a, P < 0.05), and tanshinone IIA treatment decreased the frequencies of Th17 cells significantly (Fig. 3a, P < 0.05). On the contrary, the frequencies of Treg cells showed increased accordingly, but there were no differences of the frequencies of Treg cells among the groups (Figs. 3b and 3c). The ratio of Th17/Treg cells increased in the blood of MI/RI group rats when compared to the sham group, and tanshinone IIA treatment decreased the ratio of Th17/Treg cells, but the changes has not any statistical difference between groups (Fig. 3c).

MI/RI: myocardial ischemia-reperfusion injury; *P < 0.05 vs. Sham; #P < 0.05 vs. MI/RI; IL: interleukin.

**Figure 4:**
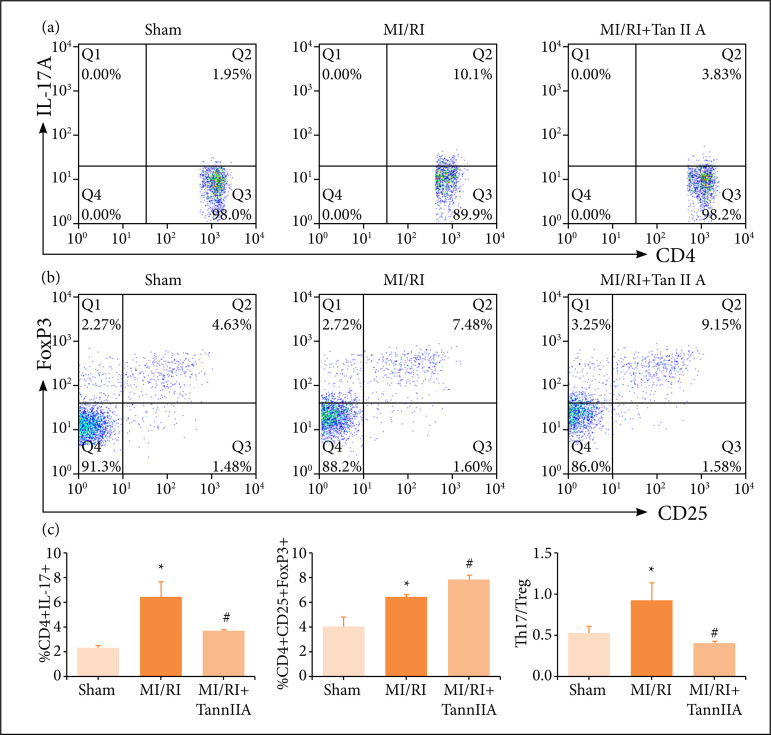
Effect of tanshinone IIA on T cells differentiation in spleen. Representative flow samples of**(a)** CD4+IL17A+ cells and **(b)** CD4+CD25+FoxP3+ cells were shown from each sample.**(c)** Quantification of cell frequency of Th17 (CD4+IL17A+), Treg(CD4+CD25+FoxP3+) and Th17/Treg (n ≥ 3).

Similarly, we measured the Th17/Treg imbalance in spleen. Flow cytometry was conducted to investigate the role of Th17 and Treg cells in the therapeutic effects of tanshinone IIA on MI/RI model. According to flow cytometric analysis (Fig. 4), the MI/RI challenge resulted in a significant increase in the proportion of Th17 (CD4+IL-17A+) cells (Figs. 4a-4c) and Treg (CD4+ CD25+ Foxp3+) cells (Figs. 4b-4c). In addition, the Th17/Treg ratio was dramatically increased in MI/RIgroup compared with their control counterparts (Fig. 4c). Moreover, the percentage of Treg cells in tanshinone IIA intervention group was elevated further (P < 0.05), whereas the proportion of Th17 cells, as well as Th17/Treg ratio, was decreased.

MI/RI: myocardial ischemia-reperfusion injury; *P < 0.05 vs. Sham; #P < 0.05 vs. MI/RI; IL: interleukin.

### Tanshinone IIA regulated CD4+ T cell differentiation via NLRP3 inflammasome

**Figure 5:**
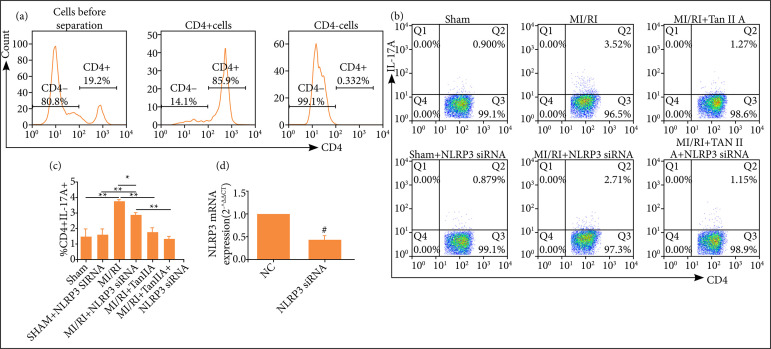
Inhibition of NLRP3 suppressed Th17 differentiation of CD4+ T cells. **(a)** Percentages of CD4+ T cells that were isolated by positive selection with MACS CD4 microbeads as shown by FCM. **(b)** The percentage of Th17 cells wasassessed by FACS. **(b)** Data were expressed as representative FACS images or **(c)** corresponding statistical graph (n ≥ 3).**(d)** The mRNA expression of NLRP3 in CD4+ T cells transfected with NLRP3 siRNA or their controls (n ≥ 3).

CD4+T cells were isolated with MACS CD4 microbeads from rat spleens and evaluated by flow cytometry. As shown in Fig. 5a, the isolated CD4+ cells were stained with CD4-PE, and the histogram displayed staining of gated viable leukocytes. In this way, we got CD4+ T cells which were high purity (> 85%). CD4+ T cells were transfected with corresponding NLRP3 siRNA or their control siRNA. By use of qPCR, the results showed that, compared with control group, the mRNA expression of NLRP3 was markedly reduced in NLRP3 siRNA group (Fig. 5d). The percentage of Th17 cells (Figs. 5b and 5c) was assessed by FACS. As shown in Figs. 5b and 5c, the percentage of Th17 cells in CD4^+^ T cells transfected with NLRP3 siRNA in MI/RI group was significantly increased than those in the sham group (Figs. 5b and 5c, P < 0.01). Tanshinone IIA treatment decreased the frequencies of Th17 cells significantly (Figs. 5b and 5c, P < 0.01). Moreover, the percentage of Th17 cells in CD4^+^ T cells transfected with NLRP3 siRNA in MI/RI group was lower than that transfected with control siRNA (Figs. 5b- and 5c, P < 0.05).

NLRP3: pyrin domain containing 3; MI/RI: myocardial ischemia-reperfusion injury; *P < 0.05; **P < 0.01; # P < 0.05 *vs*. normal control; IL: interleukin.

## Discussion

The NLRP3 inflammasome is a vital mediator of inflammatory responses, and plays a crucial part in heart diseases. In human atherosclerosis, NLRP3 inflammasome-related genes were highly expressed in atherosclerotic plaque^17^. Activation of the NLRP3 inflammasome was also associated with adverse cardiac remodeling. In transverse aortic constriction (TAC), induced mice adverse cardiac remodeling model, NLRP3 was activated in cardiomyocytes, and the activation of NLRP3 inflammasome induced increase of caspase-1 activity and production of IL-1β and IL-18^18^. MCC950, a NLRP3 inflammasome inhibitor, inhibited atherosclerotic effect, probably in a way of inhibiting the adhesion of monocytes and reducing intraplaque macrophages accumulation^19^.

Th17 cells belong to the CD4+ Th cell subgroups, which can produce IL-17A, IL-17F, IL-22 and IL-21 and participate in the body’s inflammatory response. Treg cells are CD4+CD25+ T lymphocytes that exert a negatively immune regulation function, and they secrete various immunoregulatory factors, which prevent the acute inflammatory responses of effector cells and play a critical role in maintaining autoimmunity. Th17 and Treg cells play opposite roles in immune regulation and disease occurrence. When they keep dynamic balance, they can maintain normal immune function, but, when their proportion is unbalanced, they will lead to inflammation and autoimmune diseases. IL-17 produced by Th17 led to fibrosis and ventricular remodeling in an ischemic heart failure model^20^. And the use of anti-IL-17 antibodies to block the production of IL-17 reduced the fibrosis of heart in isoproterenol-induced heart failure^21^. Treg cells inhibited proinflammatory cell infiltration and alleviated ventricular remodeling in rats of myocardial infarction^22^.

Nowadays, the interaction between Th17 and NLRP3 inflammasome, as well as the regulation of NLRP3 inflammasome on Th17 cells differentiation, was studied mainly in autoimmune diseases, infectious diseases, and inflammatory diseases, such as lupus^23^, pulmonary paracoccidioidomycosis^24^, and rheumatoid arthritis^25^. These studies showed that the NLRP3 inflammasome regulated the percentage of Th17 cells. However, data on the roles of NLRP3 inflammasome on the frequencies of Treg and Th17 cells in MI/RI are scarce.

In this study, the effects of tanshinone IIA in a rat model of MI/RI were investigated. The results showed that tanshinone IIA could improve cardiac function after MI/RI, including AV Peak Vel, EF% and FS%. The myocardial infarct size was evaluated by TTC staining, and tanshinone IIA treatment reduced the degree of myocardium injury. HE staining showed tanshinone IIA treatment group improved the myocardial injury and inflammatory infiltration. Tanshinone IIA attenuated inflammatory response and NLRP3 inflammasome activation. Tanshinone IIA decreased protein expression of NLRP3 and caspase-1, as well as the mRNA expression of NLRP3, caspase-1, IL-1β, IL-18 and FoxP3. Tanshinone IIA regulated th17/Treg cell balance in MI/RI rats. Tanshinone IIA treatment decreased the frequencies of Th17 cells in blood significantly. In spleen-derived lymphocytes, tanshinone IIA intervention increased the percentage of Treg cells, but reduced the proportion of Th17 cells, as well as Th17/Treg ratio. Moreover, the percentage of Th17 cells in CD4+ T cells isolated from MI/RI group rats that transfected with NLRP3 siRNA was reduced than CD4+ T cells transfected with control siRNA. These results indicated that th17/Treg cell differentiation was regulated by NLRP3 inflammasome.

Meanwhile, there are still limitations in this study. While tanshinone IIA regulates NLRP3 inflammasome activation and th17 cells differentiation, its mechanism and key targets need further investigation. The mechanism and precise targets of tanshinone IIA should be further studied in future work.

## Conclusions

NLRP3 inflammasome regulated the percentage of th17 cells in MI/RI. This work highlighted new mechanism of tanshinone IIA in MI/RI involving regulating of NLRP3 inflammasome activation and th17 cells differentiation, which may prove to be a potential strategy in treating MI/RI in the future.
